# Access and utilization of youth friendly sexual and reproductive health services among illiterate adolescents in Rwanda: A mixed-methods participatory study

**DOI:** 10.1371/journal.pone.0325184

**Published:** 2025-06-24

**Authors:** Madeleine Mukeshimana, Aimable Nkurunziza, Gerard Nyiringango, Eliphaz Karamage, Domina Asingizwe, Claudine Nshutiyukuri, Sandra Musabwasoni, Vedaste Bagweneza, Michael Habtu, Josephine Uzayisenga, Oluyinka Adejumo, Endale Tamrat

**Affiliations:** 1 University of Rwanda, College of Medicine and Health Sciences, Kigali, Rwanda; 2 University of Toronto, Lawrence Bloomberg Faculty of Nursing, Toronto, Ontario, Canada; 3 Western University, Faculty of Health Sciences, Arthur Labatt Family School of Nursing, London, Ontario, Canada; 4 Nipissing University, Faculty of Education and Professional Studies, School of Nursing, North Bay, Ontario, Canada; 5 Ministry of Health, Rwanda Biomedical Center/Maternal, Child and Community Health Division, Kigali, Rwanda; 6 East African Community Regional Centre of Excellence for Vaccines, Immunization, and Health Supply Chain Management, Kigali, Rwanda; 7 The Center of International Reproductive Health Training, University of Michigan (CIRHT-M), Michigan, United States of America; University of Hull, UNITED KINGDOM OF GREAT BRITAIN AND NORTHERN IRELAND

## Abstract

**Background:**

Youth Friendly Health Services Centers (YFHS) in Rwanda have been established to address the lack of information and services related to reproductive health. However, there is a paucity of studies investigating the hurdles faced by illiterate adolescents when seeking sexual and reproductive health services. This study assessed adolescents’ sexual and reproductive health (ASRH) accessibility and utilization in YFHS among illiterate adolescents in Rwanda.

**Methods:**

This study used a mixed-method participatory study design. One hundred fifty illiterate adolescents were recruited using a convenience sampling. A checklist was used to observe the 16 YFHS. In the quantitative phase, data were organized using Statistical Package for the Social Sciences (SPSS) version 26, and analyzed through descriptive and inferential statistics. Two focus group discussions moderated by trained illiterate adolescents were conducted. Data were organized using Dedoose software and analyzed thematically.

**Results:**

The proportion of YFHS utilization was 25.3%. In the multivariate regression analysis, five outcomes remained significant to utilize YFHS: ever heard about YFHS (Adjusted Odds Ratio [AOR] = 6.32; 95% Confidence Interval [CI] = 2.07–19.27, having ASRH information (AOR = 8.99; 95 CI = 1.43–56.77), having information about any family planning (AOR = 19.00; 95 CI = 1.52–236.84), use of any type of contraceptives (AOR = 4.45; 95%CI = 1.34–14.85), and having information on prevention and management (AOR = 24.99; 95 CI = 2.76–226.53). Observers rated the quality of SRHs in YFHS at 24.36%. Facilitators to access YFHS include having information about ASRH, free-of-charge services, and peer educators. The reported barriers included providers’ negative attitudes, internalized stigma, and lack of materials tailored to illiterate adolescents. The study participants suggested ways to improve the YFHS, such as community awareness, staff training, entertainment, and increasing the number of YFHS.

**Conclusions:**

The utilization of YFHS among illiterate adolescents remains low, highlighting the need for targeted interventions to improve access and quality. Key facilitators, such as access to information, peer education, and free services, should be strengthened, while addressing barriers like provider attitudes, stigma, and resource limitations. These findings underscore the necessity of enhancing both the accessibility and quality of YFHS to ensure comprehensive reproductive health care for illiterate adolescents.

## Introduction

The United Nations (UN) adopted the Sustainable Development Goals (SDGs) to recognize the importance of Health and well-being for all ages. Goal Three ensures sexual and reproductive health (SRH) for all, regardless of age, gender, or socioeconomic status and to achieve this, Target 3.7 aims to provide universal access to family planning, information, and education about SRH [1]. However, many low- and middle-income countries (LMICs) are still lagging in providing SRH services that are accessible, affordable, and of good quality [2]. This disparity particularly LMICs as they may have limited resources, cultural barriers, and societal norms. Nevertheless, as part of their efforts to ensure that sexual and reproductive health information is accessible to young people, countries have implemented youth-friendly health services (YFHS). The World Health Organization’s (WHO) 2001 Global Consultation on YFHS explains that these services have been designed with the purpose of providing safe and welcoming environments where young people can access accurate and trustworthy information about sexual and reproductive health and receive the proper services and support that they need. As outlined in the WHO guidelines, youth friendly health services should be accessible, acceptable, equitable, appropriate, and effective [3].

A growing body of research demonstrates that YFHS do not meet adolescents’ diverse and evolving needs [4–8]. In a qualitative study to assess the YFHS in Bangladesh, adolescents reported several challenges when accessing and utilizing those services, including shortages of medicine, lack of privacy, shortage of time dedicated to sexual and reproductive health services and lack of behavior change communication/educational materials [9]. Another study that was conducted in Mozambique exposed substantial deficiencies in providing information and communicating with users of SRHS [4]. A systematic review also found that YFHS in Sub-Saharan Africa are hampered most frequently by structural barriers, such as the negative attitudes of health workers and unskilled health workers, as well as individual barriers resulting from a lack of knowledge among youth [10]. However, there is a global and regional scarcity of literature examining adolescents’ access to sexual and reproductive health care, with specific focus on illiterate adolescents. Consequently, a knowledge gap exists regarding the access and utilization of YFHS by illiterate adolescents, defined as adolescents who do not know how to read and write.

According to the latest Rwanda Population and Housing Census conducted in August 2022, 16.3% of the resident population aged 15 years and above had no primary education, while the overall non-attendance rate for the population aged 3 years and above was 16.4%. This census was conducted in line with the United Nations’ recommendation to perform such surveys every ten years [11]. In this study, individuals who never attended school are classified as illiterate.

According to the World Health Organization [12], ‘Adolescents’ are individuals aged 10–19 years, ‘Youth’ refers to those aged 15–24 years, and ‘Young People’ encompasses the broader age range of 10–24 years. When using health services, people with low literacy have difficulty understanding written information, complex explanations, and instructions. Having low literacy can have a significant negative impact on clients’ interactions with healthcare workers [13]. This is a double burden for illiterate young people seeking sexual and reproductive health services. Illiterate adolescents often have difficulty accessing information about SRH and seeking related services [14–16]. In Rwanda, as of 2021, the percentage of illiterate adolescents among adolescents aged between 15–24 was 13% [17]. Despite the high rate of illiteracy among adolescents and the unique challenges they face when accessing health services, there is no single study that has looked at their access and utilization of YFHS Rwanda. Moreover, the global and regional literature on accessibility and availability of ASRH overlooks adolescents who are illiterate, a particularly high risk group. Thus, this study aims to assess the accessibility and utilization of quality sexual and reproductive health services by illiterate adolescents in both rural and urban areas in Rwanda. The study findings unveil the barriers encountered by this group of adolescents, thereby informing the policies that take into account the specific needs of illiterate adolescents when seeking SRHS.

## Methods

### Research question

What are the barriers and facilitators influencing access to and utilization of youth-friendly sexual and reproductive health services among illiterate adolescents in Rwanda?

### Study design

To shift the balance of power and gain more understanding on how illiterate adolescents access and utilize the sexual and reproductive health services, we used a participatory action research design with embedded mixed methods. The use of participatory methods in research among underrepresented youth, e.g., illiterate individuals, offers numerous advantages [18,19]. Through the use of these qualitative and quantitative methods, researchers can create an inclusive and empowering environment where individuals who cannot read and write can actively contribute to the co-creation of knowledge. Participation in research ensures cultural relevance, respect, and meaningful outcomes [20]. The use of participatory methodology can unlock the potential of illiterate individuals and produce research outcomes that are truly reflective of their realities and experiences. In this study, illiterate adolescents were involved in the study participants’ recruitment, and qualitative data collection process. The data collection instrument was validated in a consultation meeting with illiterate adolescents. Illiterate adolescents led focus group discussions (FDGs), which was a valuable approach to ensuring their voices were heard and their expertise was respected. The study findings were also validated through a workshop, where participants reviewed and discussed the research data. This participatory approach helped to ensure that research findings were relevant and applicable to illiterate adolescents.

### Setting and participants

This study was conducted in 16 YFHS in all four provinces in Rwanda and Kigali City representing rural, semi-urban, and urban areas. YFHS in Rwanda offer comprehensive information and services tailored to address the unique needs of young people, particularly in the area of ASRH. These services aim to empower adolescents and young adults with knowledge and resources to make informed decisions about their health and well-being. The ASRH-related services provided through YFHS include access to contraceptive options, counseling on family planning, prevention and management of sexually transmitted infections (STIs) including HIV, and education on safe sexual practices.

The study participants were youth who were unable to read and write. For quantitative data collection, the sample size for illiterate adolescents was calculated using single proportion formula n = z2pq/d2 where n = sample size, z statistic for a level of confidence (1.96 for 95% confidence level), p = expected prevalence or proportion, q = 1-p and d = Precision [21]. The proportion of utilization of sexual and reproductive health services was considered as 8.6% according to the recent study done on Ethiopia [22]. Based on the above formula, the sample size was 122 participants. An additional 20% was added to the calculated sample size to achieve a final total of 150 participants, ensuring sufficient statistical power and reliable results [23]. For the qualitative data collection, we exclusively interviewed adolescents who consented to participate in the study.

### Sampling, recruitment and informed consent

Data were collected from August to October, 2022. For quantitative data collection, convenience and snowball sampling were used to select 150 illiterate adolescents aged from 10 to 24 years. We employed non-probability sampling methods, as they can be particularly useful in studies involving populations that have not been extensively researched [24]. Additionally, non-probability sampling was employed due to the lack of a sampling frame for illiterate adolescents, who are also challenging to identify and reach within the community. The procedure for convenient sampling involved selecting participants who could be easily reached and were in close proximity to the data collectors. Given the existence of cooperative networks among illiterate adolescents, we employed snowball sampling, asking participants to refer others who could be potential participants. After ethical approval, the researchers requested permission from the 16 health centers that provided youth services. The permission letters from the health centers were presented to the Youth Center staff to gain access to the youth study participants. With their permission, we advertised for participants to join our study with flyers explained in YFHS. The YFHS staff members played a crucial role in explaining the flyers to potential study participants. A verbal announcement recruiting participants was also made in the monthly community work, commonly known as umuganda. Those interested in participating in the study contacted the research team members to arrange an appointment. Illiterate adolescents also brought other participants who had not heard through our advertisements.

For observations, the study team members visited and observed all 16 YFHS enrolled in the study using the observational checklist to assess the availability and quality of adolescents’ sexual and reproductive health services.

For qualitative data collection we used purposive sampling. To avoid selection bias, at the end of the quantitative survey, every participant was invited to participate in the qualitative focus groups. The research team members correctly identified the qualitative study goals along with clearly defined requirements for our target population [25]. All 150 participants who took part in the quantitative study were invited to participate in the qualitative component. However, only 18 participants agreed to take part in the focus group discussions (FGDs). As a result, we conducted two focus groups from two different study sites—one rural and one urban. According to the literature, two FGDs can capture approximately 80% of the key potential themes, making them a valuable supplement to the quantitative findings in our study [26]. Each focus group consisted of nine illiterate adolescents, with one participant at each study site serving as the moderator for the interviews.

### Data collection instruments

In the quantitative phase, we used the World Health Organization (WHO) questionnaire developed to assess accessibility/utilization of adolescent sexual and reproductive health services (ASRHS). The quantitative instrument asks about the socio-demographic characteristics of the respondents, utilization and accessibility of ASRHs, including family planning and reproductive information, and contraceptive methods used. The questionnaire also asks about factors influencing the accessibility of ASRHS by illiterate adolescents, barriers to accessing SRHS, and facilitators of YC utilization. The Checklist for the quality of YFHS was adapted from WHO’s Services Availability and Readiness Core Assessment (SARA) to measure the availability and quality of adolescent SRHS [27]. SARA is a health facility assessment tool developed to evaluate and monitor the availability and preparedness of health services. It aims to produce evidence to inform the planning and management of health systems. SARA employs a systematic survey approach to generate tracer indicators that measure service availability and readiness. Its primary goal is to provide reliable and consistent data on service delivery, including the presence of essential human resources, infrastructure, basic equipment, amenities, essential medicines, diagnostic capabilities, and the overall readiness of health facilities to deliver fundamental healthcare interventions. It was composed of the following sections: indicators of quality SRHS provided to adolescents, sexuality education services, family planning information services, safe motherhood services, post-abortion care (PAC) services, prevention and management of STIs and HIV and AIDS services. This checklist was created with sections requiring respondents to answer either YES (1) or NO (0). For the qualitative phase, a focus group discussion guide was developed by senior researchers with extensive experience in ASRHs. The interview guide was presented to the participating illiterate adolescents to gather their input. The main questions in the interview guide are outlined below. Assisted by RAs, interviewers also used additional probes based on participants’ responses and, in some cases, referred to quantitative results to guide the discussion:

1. *What do you think are the factors that hinder your access to Sexual and Reproductive Health Services (SRHS)?*
2. *What do you think are the factors that motivate you to access Sexual and Reproductive Health Services (SRHS)?*
3. *If you had the power to make changes, what would you do to make Adolescent Sexual and Reproductive Health Services (ASRHS) more available and accessible to you?*

### Data collection procedures

After gaining assent and informed consent, the research team members (SM, CN, VB, & GN) assisted the study participants, who could not read or write, in filling out the questionnaires in a private location at the study sites. The interviews were followed by an observation of the available ASRHs using an observational checklist. If they could not observe directly, the research team asked some questions to social and healthcare providers. In the qualitative phase, the study participants moderated the FGDs, assisted by one research team member who took notes. The research team members trained these moderators on the research process, data collection procedures, conducting interviews, and exercising reflexivity. They monitored the discussion to ensure it focused on the study’s topic. The FGDs were audio-recorded in Kinyarwanda, the local language that the study participants understood easily. FGDs held in a private place accessible by the participants and moderators only. The first focus group lasted 48 minutes, while the second lasted 36 minutes.

### Ethics

We received ethics approval from the University of Rwanda, College of Medicine and Health Sciences Institutional Review Board (No336/CMHS IRB/2022) on May 31st 2022. To obtain consent, since the study population was illiterate, the researchers read the information letter aloud several times to facilitate the study participants to sign informed consents (fingerprints). Participants below 18 years and their legal guardians signed assents and informed consent, respectively. Adolescents’ legal guardians are generally their biological parents. However, when the adolescent was an orphan or living independently, community consent was permitted from leaders, religious leaders, older siblings, workers in YFHS, and friends [28]. Some adolescents wanted to involve others beyond their families, such as friends. Data collectors read and explained the information letter to the legal guardians, giving them ample time to ask questions. For those aged 18 and above, they signed an informed consent. After one week, they provided their fingerprints as way of signing assents and consent forms. Data collectors allowed adolescents to speak with individuals they trusted to witness the assent and informed consent [29].

Interview audio were kept on the principal investigator’s personal device and will be destroyed according to the University of Rwanda ethical guidelines. Transcripts do not identify the participants or their demographics. Before data collection, the research team explained the purpose of the study, its benefits, that participation is voluntary, and they may withdraw at any time without any consequences. Participants signed consent forms before data collection began.

### Analysis

For the quantitative phase, Statistical Package for the Social Sciences (SPSS) version 26 was used to organize data. Descriptive and inferential statistics were used to analyze data. Frequencies and percentages were used for descriptive statistics including the demographic data of the study participants, utilization and accessibility of sexual and reproductive health services, family planning and reproductive information and contraceptive methods used, barriers to accessing YFHS, and facilitators of YFHS utilization. A bivariate analysis was conducted using the chi-squared test to determine whether dependent and independent variables were associated. The direction and strength of the factors associated with the utilization of youth friendly corner/services were determined using adjusted odds ratios along with their corresponding 95% of confidence interval (CI). In the bivariate analysis, ten significant variables (p < 0.05) were subjected to a multivariate analysis after controlling potential confounding factors; five variables were independently associated with utilization of YFHS in the final model. The model fitness was assessed using the Hosmer-Lemeshow (Chi square = 1.16; p value = 0.979), which indicates that the model was adequate. To ensure the reliability of our findings, we used standardized questionnaires developed by the WHO, provided comprehensive training to data collectors, and appointed a research coordinator who closely monitored the data collection process.

For the qualitative phase, data were organized using Dedoose software and analyzed thematically following six phases: data familiarization; coding; generating themes; reviewing themes; defining and naming themes; and reporting [30–32]. Three research team members agreed on codes and themes. For data familiarization, three research team members assisted with data collection and transcribed and translated the data. Three research members also participated in the initial coding, which involved carefully reviewing the data and identifying recurring patterns and themes. The researchers used inductive and deductive coding to create a comprehensive coding framework. After developing the initial set of codes, the researchers discussed the developed code with the whole research team members and began data reduction. This involved organizing and condensing the codes into fewer themes or categories. The researcher reviewed the coded data, compared and contrasted codes, and identified patterns across different data sources. The research team members examined the relationships between the themes and drew conclusions based on the data. The research team members used a combination of reflection, interpretation, and critical thinking skills to interpret the data in a meaningful way. The research team held several meetings to derive themes from the data. The research team members reviewed and refined the themes identified, ensuring that they were grounded in the data and made sense in the context of the overall research question. The themes were discussed and agreed upon with other research team members to ensure credibility and validity. After conducting data analysis, the participants were invited to a workshop to validate the findings. During the workshop, the participants reviewed the analysis results, discussed their implications, and offered their insights and suggestions. Triangulation was employed to address and reconcile discrepancies between the quantitative data and the qualitative data.

## Findings

### Quantitative findings

#### Socio-demographic characteristics of the illiterate adolescents in Rwanda, 2023.

The respondents’ average age was 19.5 years, and about half (49.3%; n = 74) were between the ages of 15 and 19 years. The majority of them (66.0%; n = 99) were females, and 66.7% (n = 100) of them lived in rural areas. Almost all (95.3%; n = 143) of them were single, and 88.7% (n = 133) indicated that they were still living with their parents. Christianity predominated in terms of religion (92.7%; n = 139). When asked about their monthly income, the majority (72.0%; n = 108) of the illiterate adolescents reported 5000 Rwandan Francs or less (Table 1).

**Table 1:** Socio-demographic characteristics among illiterate adolescents in Rwanda, 2023.

Variables	N = 150	%
**Age [years]**		
10–14	5	3.3
15–19	74	49.3
20–24	71	47.3
Mean(SD) = 19.5(2.92)		
**Gender**		
Female	99	66.0
Male	51	34.0
**Location**		
Rural	100	66.7
Semi-urban^*^	44	29.3
Urban	6	4.0
**Living Status**		
Alone	6	4.0
Friends	6	4.0
Husband	5	3.3
With parents	133	88.7
**Marital Status**		
Married	7	4.7
Single	143	95.3
**Religion**		
Christianity	139	92.7
Islam	6	4.0
Other	5	3.3
**Monthly Income** ^**^		
> 20000 RWF/Month	5	3.3
6000-20000 RWF/Month	37	24.7
5000 RWF and below/Month	108	72.0

* Semi-urban in our study is defined as a transitional zone between rural and urban settings characterized by moderate population density, moderate development of infrastructure and with both agricultural and small scale industrial activities.

** Female adolescents have an average monthly income of 41,651 Frws, while their male counterparts earn an average of 72,165 Frws per month [33].

### Accessibility and utilization of YFHS

The majority of respondents (54.7%; n = 82) claimed to have never heard of YFHS. Among those who ever heard of YFHS (45.3%, n = 68), 45.6%, n = 31 indicated a walking distance of 30 minutes to 1-hour to the YFHS, and 55.9%, n = 38 were aware of the services provided at the YFHS. However, more than half (53.3%; n = 80) had information about ASRHS and the main source of information was through social media and friends (33.8%; n = 27). The proportion of ever YFHS utilization, was 25.3% (n = 38), where most of them reported that the waiting time was reasonable (26%, n = 68.4%), the operating hours were appropriate (94.7%, n = 36), and the services were free of charge (84.2%, n = 32). The most common services received during the YFHS visit were education on the dangers of premarital and unsafe sex (73.7%, n = 28), education on human biology (63.2%, n = 24) as well as education on puberty and menstrual hygiene practices (63.2%, n = 24) (Table 2).

**Table 2:** Utilization and accessibility of YFHS among illiterate youth in Rwanda, 2023.

Variables	N = 150	%
**Ever heard about YFHS**		
No	82	54.7
Yes	68	45.3
**Walking time to YFHS (n = 68)**		
1 h–2 hours	10	14.7
30 min − 1 hour	31	45.6
Less than 30 min	27	39.7
**Knowing any kind of services that YFHS provide to adolescents and youth (n = 68)**		
No	30	44.1
Yes	38	55.9
Having i**nformation about ASRHS**		
No	70	46.7
Yes	80	53.3
**How the information was obtained (n = 80)**		
Health education (Campaigns/awareness) Time for interaction with health care provider	9	11.3
Time for interaction with health care provider Community Health Workers	6	7.5
Via friends Time for interaction with health care provider Community Health Workers	4	5.0
Via social media Time for interaction with health care provider Community Health Workers	10	12.5
Via social media and via friends	27	33.8
Via social media, Via friends and Community Health Workers	7	8.8
Via social media, Via friends and Health education (Campaigns/awareness) Time for interaction with health care provider	8	10.0
Via social media, Via friends and Time for interaction with health care provider Community Health Workers	9	11.3
**Ever visited the YFHS**		
No	112	74.7
Yes	38	25.3
**Waiting time for those visited YFHS (n = 38)**		
Long (30-1h)	11	28.9
Reasonable (<30min)	26	68.4
Very long (>1h)	1	2.6
**Suitability of operational hours for those YFHS (n = 38)**		
Not suitable	1	2.6
Suitable	36	94.7
Very suitable	1	2.6
**The cost of services for those visited YFHS (n = 38)**		
No cost	32	84.2
Reasonable cost	6	15.8
**Service received from the YFHS during the visit (n = 38)**		
Education on human biology	24	63.2
Education on puberty and menstrual hygiene practices	24	63.2
Education on healthy associations	14	36.8
Education on dangers of premarital and unsafe sex	28	73.7
Information on reproductive rights and policy	15	39.5
Information on harmful cultural practices like female circumcision	11	28.9
Information on prevention of non-infectious conditions of reproductive health such as fistula and cancers	13	34.2

### Family planning and reproductive information and contraceptive methods used

As indicated in Table 3, 43.3% (n = 65) had information about any type of family planning methods. Male condoms were mentioned as one of the primary family methods (42.7% (n = 64). With regard to contraceptive use, 27.3% (n = 41) of the respondents (both males and females) reported they had ever used any type of contraceptives. Considerable percentages had knowledge on voluntary counseling and testing (38.7%; n = 58), as well as prevention and management of STIs and HIV (42.0; n = 63). However, only 11.3% (n = 17) and 10.0% (n = 15), respectively, had knowledge of antiretroviral therapy and mother-to-child transmission prevention.

**Table 3:** Family planning, reproductive information, and contraceptive methods used among illiterate youth in Rwanda, 2023.

Variables	Yes, n(%)	No, n(%)
Information on oral pills	54(36.0)	96(64.0)
Information on Male condoms	64(42.7)	86(57.3)
Information on Female condoms	31(20.7)	119(79.3)
Information on intrauterine contraceptive device	44(29.3)	106(70.7)
Information on emergency contraceptives	48(32.0)	102(68.0)
Information on implants contraceptives	52(34.7)	98(65.3)
Having information on any type of family planning	65(43.3)	85(56.7)
Use of any type of contraceptives among males and females	41(27.3)	109(72.7)
Use of oral pills among females (N = 99)	3(3.0)	96(97.0)
Use of injectable contraceptives (N = 99)	7(7.1)	92(92.9)
Use of female condoms (N = 99)	1(1.0)	98(99.0)
Use of intrauterine contraceptive device (N = 99)	0(0.0)	99(100.0)
Use of emergency contraceptives (N = 99)	2(2.0)	97(98.0)
Use of implant contraceptives (N = 99)	17(17.2)	82(82.8)
Use of male condoms (N = 51)	8(15.4)	44(84.6)
Information on prevention and management of STIs, HIV and AIDS	63(42.0)	87(58.0)
Information on voluntary counseling and testing	58(38.7)	92(61.3)
Information on antiretroviral therapy	17(11.3)	133(88.7)
Information on services for the prevention of mother-to-child transmission of HIV and other STIs	15(10.0)	135(90.0)

### Barriers to accessing SRHS and facilitators of YFHS utilization

The key barriers identified were lack of awareness of sexual and reproductive health services (46.0%; n = 69), unable to afford to access the SRHS (28.7%; n = 43), afraid of being viewed negatively by the community because of the use of SRHS when young (35.3%; n = 53) and being unable to read and write (52.0%; n = 78). On the other hand, getting family encouragement (57.3%; n = 86), getting a friend’s encouragement (69.3%; n = 104), staff being kind at the youth friendly services (58.7%; n = 88), and social media campaigns (65.3%; n = 98) were found to be the motivators of using youth friendly services corner for SRHS (Table 4).

**Table 4:** Barriers to accessing SRHS and facilitators of youth-friendly services utilization among illiterate youth in Rwanda, 2023.

Variables	Yes, n(%)	No, n(%)
**Situations that may be barriers to you for accessing the SRHS**		
My religious beliefs	24(16.0)	126(84.0)
My family (My family does not like me to use the SRHS at my age)	25(16.7)	125(83.3)
I don’t know that SRHS exist	69(46.0)	81(54.0)
I don’t have money to access the SRHS	43(28.7)	107(71.3)
I don’t have time to visit the services (I am too busy)	5(3.3)	145(96.7)
I don’t want the society/community to see me as a prostitute because use SRHS at my age	53(35.3)	97(64.7)
I don’t visit the services because I think I will not be able to know how to use them as I don’t know how to read and write	78(52.0)	72(48.0)
The staff at the youth friendly services/corners are not kind	1(0.7)	149(99.3)
**Situations that may motivate you to visit the YFHS for SRHS**		
My family encourages me	86(57.3)	64(42.7)
My friends encourage me	104(69.3)	46(30.7)
The staff at the Youth friendly services/corners are kind	88(58.7)	62(41.3)
I get motivated because of regular campaigns about ASRHS by health care providers	51(34.0)	99(66.0)
I get motivated through social media campaigns	98(65.3)	52(34.7)
I am motivated through our regular youth association’s information about SRHS	29(19.3)	121(80.7)

### Factors associated with utilization of YFHS

Those who ever heard about youth friendly services/corners were 6.3 time more likely to utilize YFHS (AOR = 6.32; 95%CI = 2.07–19.27. The odds of using YFHS was about nine times among respondents with information on ASRHS (AOR = 8.99; 95 CI = 1.43–56.77). The odds of utilizing YFHS was 4.4 times among those using any type of contraceptives (AOR = 4.45; 95%CI = 1.34–14.85). Respondents with information on prevention and management of STIs were about 25 times more likely use YFHS (AOR = 24.99; 95 CI = 2.76–226.53). However, those having information on any type of family planning 0.05 times less likely to use YFHS (AOR = 0.05; 95 CI = 0.004–0.066) (Table 5).

**Table 5:** Factors associated with utilization of YFHS among illiterate youth in Rwanda, 2023.

Variables	Youth friendly services utilization		COR(95%CI)	AOR(95%CI)
	Yes, n(%)	No, n(%)		
**Age [years]**				
				
<20	8(10.1)	71(89.9)	Ref	**Ref**
20–24	30(42.30)	41(57.7)	6.49(2.72-15.49)^***^	3.26(0.86-12.26)
**Gender**				
Female	24(24.2)	75(75.8)	Ref	
Male	14(27.5)	37(72.5)	1.18(0.55-2.55)	
**Location**				
Rural	22(22.0)	78(78.0)	Ref	
Urban/Semi-urban	16(32.0)	34(68.0)	1.67(0.78-3.57)	
				
**Living Status**				
Alone	1(16.7)	5(83.3)	Ref	
Friends	1(16.7)	5(83.3)	1.00(0.05-20.83)	
Husband	3(60.0)	2(40.0)	7.50(0.46-122.69)	
With parents	33(24.8)	100(75.2)	1.65(0.18-14.64)	
**Marital Status**				
Married	3(42.9)	4(57.1)	Ref	
Single	35(24.5)	108(75.5)	0.43(0.09-2.03)	
**Religion**				
Christianity	35(25.2)	104(74.8)	Ref	
Islam	1(16.7)	5(83.3)	0.59(0.07-5.26)	
Other	2(40.0)	3(60.0)	1.98(0.32-12.35)	
**Monthly Income in Rwandan Franc**				
> 5000	20(47.6)	22(52.4)	4.54(2.06-10.01)^***^	1.49(0.48-4.64)
				
5000 and below	18(16.7)	90(83.3)	Ref	Ref
**Ever heard about youth friendly services/corners**				
No	8(9.8)	74(90.2)	Ref	Ref
Yes	30(44.1)	38(55.9)	**7.30(3.05-17.47)** ^***^	**6.32(2.07-19.27)** ^**^
**Information about ASRHS**				
No	3(4.3)	67(95.7)	Ref	Ref
Yes	35(43.8)	45(56.3)	**17.37(5.03-59.91)** ^***^	**8.99(1.43-56.77)** ^*^
**Having information on any type of family planning**				
No	8(9.4)	77(90.6)	Ref	Ref
Yes	30(46.2)	35(53.8)	**8.25(3.43-19.82)** ^***^	**0.05(0.004-0.66)** ^*^
**Use of any type of contraceptives**				
No	16(14.7)	93(85.3)	Ref	Ref
Yes	22(53.70)	19(46.30)	**6.73(2.99-15.15)** ^***^	**4.45(1.34-14.85)** ^*^
**Information on prevention and management of STIs, HIV, and AIDS**				
No	6(6.9)	81(93.1)	Ref	Ref
Yes	32(50.8)	31(49.2)	**13.94(5.31-36.58)** ^***^	**24.99(2.76-226.53)** ^**^
**Information on voluntary counseling and testing**				
No	8(8.7)	84(91.3)	Ref	Ref
Yes	30(51.7)	28(48.3)	**11.25(4.62-27.38)** ^***^	0.85(0.07-10.38)
**Information on antiretroviral therapy**				
No	28(21.1)	105(78.9)	Ref	Ref
Yes	10(58.8)	7(41.2)	**5.36(1.87-15.34)** ^**^	0.84(0.14-4.85)
**Information on services for the prevention of mother-to-child transmission of HIV and other STIs**				
No	28(20.7)	107(79.3)	Ref	Ref
Yes	10(66.7)	5(33.3)	**7.64(2.42-24.17)** ^**^	3.11(0.45-21.24)

*** p  <  0.001,

** p  <  0.01,

* p  <  0.05; COR = Crude Odds Ratio; AOR = Adjusted Odds Ratio; CI Confidence interval; Ref: Reference group.

### Observations: Availability of quality adolescent sexual reproductive health services

Observers rated the quality of SRHs in YFHS at 24.36%. Only 31.25% (n = 5) of YFHS used the written guidelines, 68.75% (n = 11) were not confidentially accessible, 68.75% (n = 11) did not have private rooms, 75% (n = 12) did have comfortable waiting areas, only 12.5% (n = 2) had strategized how to involve adolescents in feedback provision on offered services, 37.5% (n = 6) involved peer educators, and 62.5% (n = 10) failed to meet the adolescent needs according to our study’s assessment. Sexuality education services were at the level of 81.6%. All centers 100% (n = 16) could deliver family planning information services, and all family planning methods were available to adolescents. Only 12.5% (n = 2) did not have trained midwives regarding safe motherhood services. According to the PAC services, 75% (n = 12) could respond well to emergency cases of bleeding and shock. However, 93.75% of centers did not have trained PAC providers or manual vacuum aspiration (evacuation) of retained products of conception. All health centers 100% (n = 16) offered comprehensive information on preventing and managing STIs and HIV and AIDS.

### Qualitative findings

#### Facilitators to access YFHS.

Participants described the reported facilitators to access YFHS were having information about these SRH services, free of charge services, and peer educators.

Having information of ASRHS determined the accessibility and utilization of YFHS. Some participants who accessed the YFHS explained that these services are convenient and they can access them whenever they need them. For instance, one participant said, *“To get these services is very easy and they answer our questions whenever we ask them [staff]. Knowing that I have a Sexual Reproductive Health question I run to them”*. R5, FGD1 Other participants who had to travel to access services mentioned that, even though the YFHS might be far from their homes, they arrange to get sexual and reproductive health information when they are in need. For example, one participant noted*, “It’s a challenge to get to this center. It requires to have transport money which is somehow a problem. But because I know my questions will be answered I try my best to come.”* R4, FGD1. Interestingly those who have not accessed these services did not have prior information about sexual and reproductive health and the existence of these services. One participant explained, *“We don’t know these services and no one informed us about them either our parents or the leaders of our community So, how could I go there?”* R2, FGD2

A few participants reported being motivated to use these services because they are free of charge. For example, one study participant mentioned, *“I usually go there because these services are free of charge. We don’t pay anything.”* R3, FGD1. Those in the focus groups who had not accessed the services previously learned that they are free of charge, and said they will now want to use them. As one participant stated, *“For now I know that they are free of charge. That’s interesting! Definitely I will have to go there and see what they have”*. R4, FGD2 Some participants reported that they use these services because sometimes there are peer educators. For instance, one participant said, *“It is very easy to ask questions because these guys are our age. We are comfortable asking the questions.”* R2, FGD1

### Barriers to accessing and utilizing YFHS for illiterate adolescents

The study participants, adolescents who could not read, reported that they experienced barriers accessing and utilizing YFHS including: providers’ attitudes, internalized stigma, and a lack of materials tailored to their needs.

Providers’ attitudes influence how illiterate adolescents utilize YFHS. Some study participants reported that some healthcare providers were judgmental, and this kept them from going back or asking questions. For example, one participant noted, *“Some providers are not good. Sometimes they don’t give us good services. They can form some opinions before you finish to ask questions.”* R5, FGD2 Other participants questioned the confidentiality of providers at the YFHS as expressed by this participant, *“It does exist that there are providers we don’t trust because they cannot keep our information confidential…. sometimes they are friends of your parents and they immediately tell them what you shared with them.”* R7, FGD1

Stigma is another reported challenge when accessing YFHS. A few participants showed how they had internalized the stigma associated with seeking reproductive health services when they explained how they avoid any chance of being seen there. A study participant noted, *“I feel shy. I cannot go there. Imagine seeing me going there.!”* R1, FGD2 Other participants expressed that when they talk with their friends about these services, and how to access them, their friends stigmatize them. For instance, a study participant explained, *“Sometimes I can seek advice from my friends that I want to come to these services and they tell me “you are prostitute, you are this and this”. So, I immediately shun these services.”* R7, FGD1

The study participants highlighted that there is lack of materials tailored to illiterate adolescents’ needs. Most study participants explained that in these youth friendly health services there are books, flyers, and posts which all require reading. For example, one study participant said, *“Oh yeah… it’s funny! I can’t read these educational materials. It’s hard and you feel like not going back there.”* R5, FGD1 Another participant noted, “I’m not sure if, before establishing these services, consideration was given to those who are unable to read the provided materials.” R6, FGD1 The following quote illustrates how the study participants appreciate online mobile applications for sexual and reproductive health but can’t make use of them since they cannot read, *“These applications are good for those who can read but for us you cannot use them.”* R2, FGD1

### Participant recommendations to improve YFHS for illiterate adolescents

In the focus group discussions study participants suggested ways to improve the YFHS for illiterate adolescents. These ways include community awareness, staff training, entertainment, and increasing the number of YFHS.

Some participants suggested that illiterate adolescents do not utilize YFHS because they are not aware of the services offered. To address this, they suggested conducting community campaigns to increase the level of awareness. This participant noted, *“I don’t know these services and I have never heard anyone talking about them. This means there are others like me out there. Why wouldn’t you take your come here one day and teach us about them?”* R6, FGD2 Another participant added, *“Hmm… forming groups to down to the cells to teach youth. Just making the aware of what is happening in these services.”* R5, FGD1

Several participants suggested more training for health center staff since when they go there some staff do not provide enough information to their questions. For example, one participant stated, *“I would suggest to find a way of training the staff so that they give us enough information to our questions.”* R3, FGD1 Another participant added, *“ eeh…. What can I say? At times, the information provided by the workers here may vary, and I believe there is a need for better consistency and alignment in what they communicate to us ….hmm may be training, right?”* R4, FGD2

To increase the number of illiterate adolescents attending the YFHS, most participants suggested equipping these centers with entertainment equipment and activities. One participant said, *“If there are means, it would be better to have some TVs here. I know many would attend to see some music and movies and then they meet with the staff. I know some can be interested.”* R2, FGD1. Another participant confirmed this, *“What if they organize games like football? We can go there.”* R7, FGD2

Most study participants highlighted that YFHS offers much needed services and that, if they opened health centers in remote areas, more illiterate adolescents would use them. For instance, one study participant said, *“Many illiterate youths are in villages and you will be finding that these health centers are located in towns. So, it’s better to bring more YFHS so that we don’t travel that long.”* R3, FGD1

## Discussion

This study aimed to assess the accessibility and utilization of quality SRH in YFHS by illiterate adolescents in both rural and urban areas in Rwanda.

Overall, the results indicated that the SRH services are readily accessible considering that the majority responded that an adolescent walks only 1 hour or less to access YFHS, also the services are free of charge and it was found that some healthcare providers are friendly. The findings also revealed that the waiting time for services was acceptable and within a manageable timeframe.

Despite this accessibility, the current study revealed low utilization of YFHS. The current utilization rate is comparatively higher than that of a study conducted in Ghana, 7.9% [34] and lower than the one reported in Nepal, 67.05% [35] and one conducted in Uganda, 42% [36]. However, the current findings are closely aligned with those found in the study conducted in Ethiopia, 28.8% [37]. The low utilization rate in the current study may be due to the study participants’ illiteracy and the inclusion of a significant number of adolescents from rural areas. As a result, they have little to no knowledge about SRH. To close knowledge gaps and increase service utilization, targeted education and outreach campaigns are vital for enhancing SRH awareness among illiterate adolescents especially in rural areas.

Similarly to the qualitative findings, the majority of the respondents who visited the youth friendly health service reported that the waiting time was reasonable, the operating time was appropriate, and the services were free of charge. In contrast, according to a systematic review in sub-Saharan African countries, operational barriers, such as inconvenient operating hours and high costs, hinder access and use of youth friendly services [10]. Understandably, reducing waiting time, extending operating hours, and providing free services are all effective strategies to help illiterate adolescents obtain SRH services. The current results revealed that ever heard about youth friendly services/corners, having prior information on ASRHS, having information about any type of family planning, using any type of contraceptives, and seeking information on prevention and management of STIs are statistically significantly associated with accessing and utilizing YFHS. These findings corroborate a study conducted in Ghana [34], Uganda [36], and Ethiopia [37] where similar factors were reported. Raising community awareness of youth-friendly services can increase access and utilization. These findings may be attributed to the fact that increased familiarity with YFHS and prior knowledge of ASRHS enhance adolescents’ confidence and ability to access available services. Additionally, knowledge of family planning methods and STI prevention serves as a practical incentive to seek and utilize these resources effectively. Consequently, policymakers need to invest in thorough education and awareness campaigns regarding YFHS, ensuring that adolescents have the essential knowledge to effectively make use of sexual and reproductive health services.

In this study participants reported barriers in accessing and utilizing YFHS, including the negative attitudes of healthcare providers, judgmental care, and lack of confidentiality. These findings were similar to reports in other settings in Sub Saharan Africa [10]. In contrast, the findings from our observations in this study revealed that most staff were non-judgmental, friendly, welcoming, good listeners, expressed respect when interacting with adolescents, and respected privacy when providing services. The disconnection between the study participants’ narratives and the findings from observations might be related to the Hawthorne effect. This occurs when study participants know that they are being observed and often change their behavior [38]. Thus, to control for this possibility, future researchers should consider using different approaches during observations of YFHS such as unobtrusive (nonreactive) and habituation or desensitization [38].

Qualitative findings revealed that internalized stigma as a barrier to accessing sexual and reproductive care at the YFHS. This aligns with a systematic review from Sub Saharan Africa [10]. Creating an inclusive and supportive environment for illiterate adolescents can be achieved by promoting awareness, sensitizing service providers, improving communication channels, and establishing peer support networks. Our study participants also reported a lack of materials tailored to illiterate adolescents’ needs. The available educational materials and mobile applications are designed for those who can read. As reported elsewhere, illiterate adolescents often have difficulty accessing information about sexual and reproductive health and seeking services related to either of these topics [15,16]. To bridge the existing gap and provide equal opportunities to illiterate adolescents to make informed SRH decisions, innovative approaches are needed, such as multimedia tools, community-based interventions, and mobile technology tailored to their needs.

### Strengths and limitations

This study has several strengths. First, this study triangulated different data sources, including questionnaire, observations, and focus groups to gain a deeper understanding. Second, we used a participatory approach to co-create knowledge with illiterate adolescents and unpack YFHS realities. Third, our participants represented both rural and urban communities located at 16 study sites in every province of the country. So, the findings are generalizable to the Rwandan context. Fourth, using convenience and snowball sampling allowed us to expand our sample and tap into networks that may not have been easily accessible through a single sampling strategy.

However, certain limitations of this study should be considered when interpreting the findings. While assumptions were taken into account when determining the sample size, the limited sample size posed a challenge when calculating the 95% confidence interval for the odds ratios. The small sample size resulted in wide ranges for the 95% confidence intervals of the adjusted odds ratios. Consequently, the results should be interpreted with caution, considering the limitations of the sample size and the broad confidence intervals. Second, although snowball and convenience sampling can introduce sampling bias, we minimized potential errors by employing diverse recruitment strategies, including engagement with community organizations and peer networks. Furthermore, we strengthened the credibility of our findings by using methodological triangulation, incorporating qualitative data to offer a more in-depth and comprehensive understanding of the experiences of illiterate adolescents. Third, we only used two focus group discussions, which could generate 80% of the themes. This limited number of discussions may have restricted the breadth of perspectives and experiences represented, potentially missing less common or nuanced viewpoints. Fourth, some participants who refused to participate in the FGDs might have had different perspectives on YFHS use and access. Fifth, the use of binary (Yes/No) responses limited our understanding of the complexity and nuances of the barriers and facilitators by restricting participants’ ability to elaborate on their experiences. This simplistic approach risked oversimplifying intricate issues and failing to capture the depth of participants’ perspectives. To address this limitation, we employed methodological triangulation, incorporating observations and FGDs. These additional approaches provided rich, contextual insights that complemented the quantitative data, allowing for a more comprehensive and nuanced interpretation. Lastly, providers knew they were being observed during the observations, which could lead to the Hawthorne effect. Our study did not consider providers who receive illiterate adolescents. Future research should use different approaches to prevent the Hawthorne effect, including unobtrusive and habituation techniques. Further research should also involve providers to understand their perspectives when receiving illiterate adolescents.

## Conclusion

YFHS access and utilization were very low among illiterate adolescents. Factors associated with access and utilization were hearing about YFHS, having prior information on ASRHS, having information about family planning, using contraceptives, seeking information on prevention and management of STIs, having free-of-charge services, and using peer educators. Some barriers to service use and access were providers’ negative attitudes, internalized stigma, and lack of materials tailored to illiterate adolescents’ needs. The government and its SRH partners need to build on those factors identified here that lead to service access and use, including through community outreach and involving illiterate adolescents in designing and providing feedback on service provision. In addition, the government should fund the free services and private spaces for adolescents through YFHS. To address the barriers to accessing and utilizing YFHS, the government’s top priorities should be community campaigns to reach illiterate adolescents, training for providers, and developing tailored non text-based education materials.

### Study Implications

The findings of this study have significant implications for enhancing access to and utilization of SRH services among illiterate adolescents in Rwanda. Although these services are geographically and financially accessible, the low utilization rates underscore the need for targeted interventions to bridge knowledge gaps and raise awareness. Expanding education and outreach initiatives, particularly in rural areas, is essential to equipping adolescents with the information needed to make informed SRH decisions. Strengthening community engagement through awareness campaigns can help shift societal perceptions, reduce stigma, and promote the uptake of YFHS. Moreover, ensuring that adolescents receive prior exposure to information on family planning, STIs prevention, and available SRH services can instill confidence in seeking care. Policymakers and healthcare practitioners should prioritize comprehensive SRH education tailored to the needs of illiterate adolescents while exploring alternative outreach methods such as peer-led education and community-based programs.

Additionally, addressing barriers related to healthcare providers is crucial for fostering a supportive environment where adolescents feel safe and encouraged to seek services. While observational findings suggested that most healthcare providers were welcoming and respectful, participant accounts highlighted concerns about judgmental attitudes and breaches of confidentiality, likely driven by stigma and fear of discrimination. This highlights the importance of ongoing training and sensitization programs for healthcare providers to ensure the delivery of adolescent-friendly, nonjudgmental care. Furthermore, innovative strategies should be employed to make SRH information more accessible to illiterate adolescents, including the use of audiovisual content, community radio broadcasts, and interactive mobile technologies. Combating internalized stigma and service-related obstacles requires a comprehensive approach that enhances provider communication skills, establishes peer support networks, and fosters inclusive healthcare environments.
